# Beta zone parapapillary atrophy in elderly Chinese

**DOI:** 10.1186/s12886-022-02651-0

**Published:** 2022-11-11

**Authors:** Ling Xiao Zhou, Lei Shao, Wen Da Zhou, Liang Xu, Rong Li, Wen Bin Wei

**Affiliations:** 1 Department of Ophthalmology, The First Affiliated Hospital of Xi’an Medical University, Xi’an, Shaanxi China; 2 Beijing Tongren Eye Center, Beijing Key Laboratory of Intraocular Tumor Diagnosis and Treatment, Beijing Tongren Hospital, Capital Medical University, 1 Dong Jiao Min Xiang, Dong Cheng District, Beijing, 100730 China; 3 Beijing Institute of Ophthalmology, Beijing Tongren Hospital, Capital Medical University, Beijing, China

**Keywords:** Beta zone, The Beijing eye study 2011, Colour optic disc photographs, Morphometrically analyze

## Abstract

**Purpose:**

Assess the beta zone parapapillary atrophy in elderly Chinese.

**Patients and methods:**

The Beijing Eye Study 2011 is a population-based cross-sectional study, which includes 3468 patients with the average age of 64.5 ± 9.8 years. The beta zone of parapapillary atrophy was captured and analyzed morphometrically by using colour optic disc photographs.

**Results:**

The beta zone was found in 1358 (39.9%) eyes, measuring 0.37 ± 0.84 mm^2^ in size, 203.5 ± 81.8° in circumferential angle, 0.36 ± 0.27 mm in the maximum radial extent, the most often and longest in the temporal peripapillary region, followed by the temporal inferior region and the temporal superior region, the nasal region at least. Beta zone has statistically significant association with male gender (*P* = 0.001), myopic refractive error (*P* = 0.003), thinner retinal nerve fiber layer thickness (*P*<0.001), thinner subfoveal choroidal thickness (*P*<0.001), bigger size of optic disc size (*P*<0.001). The size of beta zone has statistically significant association with longer axial length (*P* = 0.004)，increasing age (*P*<0.001), urban (*P* = 0.025), cardiovascular disease history (*P* = 0.025), with age related macular degeneration (*P* = 0.038), myopic ametropia (*P*<0.001), thinner retinal nerve fiber layer thickness (*P* = 0.001), thinner subfoveal choroidal thickness (*P*<0.001), bigger size of optic disc size (*P* = 0.001).

**Conclusion:**

The population prevalence of beta zone was 39.9% in elderly Chinese. The area of the beta zone has statistically significant association with age, urban, the thickness of retinal nerve fiber layer, age related macular degeneration, cardiovascular disease history, axial length, myopic refractive error, size of optic disc size, the thickness of subfoveal choroid.

## Introduction

Elschnig found that the patients with glaucoma often had chorioretinal atrophy area around optic disc in the early twentieth century [1]. With the development of modern imaging technologies and ophthalmology, some scholars divided PPA into two parts: alpha zone and beta zone, the definition of alpha zone is irregular hypopigmentation and hyperpigmentation of the retinal pigment epithelium (RPE), beta zone lies in the area next to the disc and appears as exposed sclera and choroidal macrovascular with complete RPE atrophy [2, 3]. The experts have assessed the beta zone and investigated related factors in previous hospital-based studies and the population-based studies [4–6]. The Beijing Eye Study was a population-based prospective cohort study with five-year follow-up. The population prevalence of the beta zone was 20.95% in 2001, and the position and size of beta zone related to age, refractive error, optic disc area and best corrected visual acuity, was basically consistent with the research results from other population-based studies [7]. Five years later, the progress rate of beta zone was 8.2 ± 0.5%, and age, hypertension, high myopia, corneal thickness and glaucoma were the related factors in 2006 [8]. The current study is the third assessment of the beta zone in elderly Chinese.

## Material and methods

### Ethics statement

The data are from The Beijing Eye Study 2011. The Beijing Eye Study was a population-based prospective cohort study in Northern China. The protocol was approved by the Medical Ethics Committee of the Beijing Tongren Hospital, all investigations conformed to the tenets of the Declaration of Helsinki, and all participants gave informed written consent. The study was applied in 5 communities in the urban area and 3 communities in rural area of Beijing in 2001 and followed in 2006 and 2011, respectively. People over the age of 50 were included into the Beijing Eye Study 2011. Some literatures reported this study in detail [9–12].

### Study design and patients

The current study was part of the cross-sectional Beijing Eye Study 2011. An interview with standardized questions and systemic examinations is implemented in all participants. The questions were about demographic variables, socioeconomic background, and known major systemic diseases; the systemic examinations included a fasting blood test of blood lipids, glucose and glycosylated hemoglobin HbA1c, blood pressure, body height and weight and the circumference of the waist and hip. All participants underwent comprehensive ophthalmic examination by the trained ophthalmologists, such as visual acuity assessment, intraocular pressure (CT-60 computed tonometer, Topcon Ltd., Tokyo, Japan), slit-lamp examination of the external eye and anterior segment, and photography of the lens (Neitz CT-R camera, Neitz Instruments Co., Tokyo, Japan) and macula and optic disc (fundus camera Type CR6-45NM, Canon Inc. U.S.A.). The visual field examinations were performed by frequency- doubling perimetry using the screening program C-20-1 (Zeiss- Humphrey, Dublin, California, USA). The spectral domain optical coherence tomography (SD-OCT; Spectralis®, Wavelength: 870 nm; Heidelberg Engineering Co., Heidelberg, Germany) with enhanced depth imaging (EDI) was first performed on the participants in this time.

**Fig. 1:**
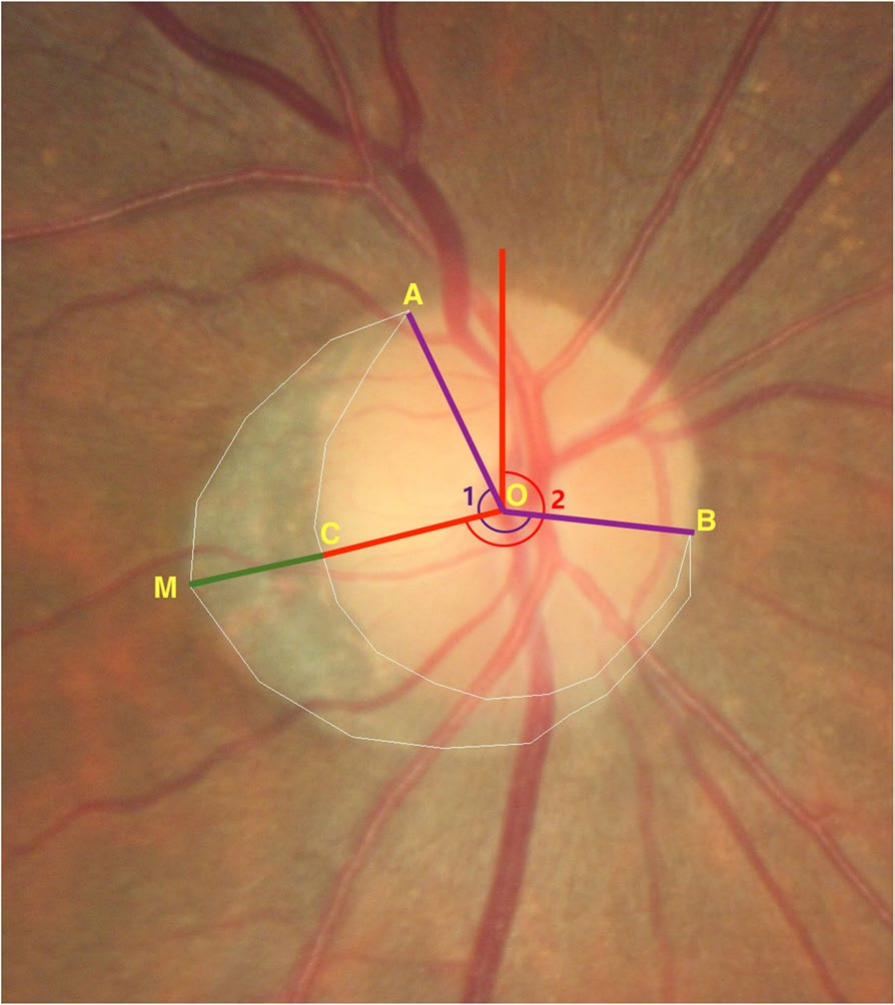
Topographic measurement of the beta zone. In the figure, point O is the center of the disc, and point A and B is the vertices of the beta zone, the point M and C is the maximum radial extent on the beta zone margin and disc margin. The beta zone is the area inside the white line, circumferential angle of the beta zone was defined as the angle between point A and B (angle 1), location of the beta zone was defined as the angle between maximum radial extent and vertical line from the center point of disc (angle 2), distance from C to M was defined as maximum radial extent of the beta zone

**Fig. 2:**
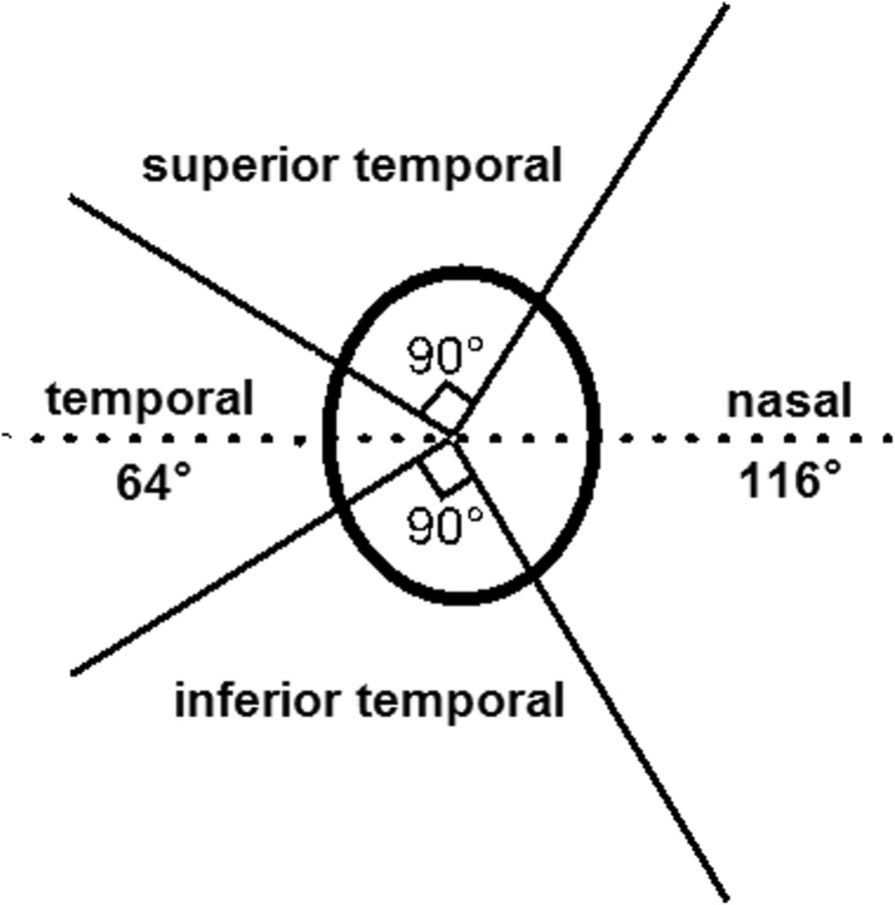
Sectors of optic disc and parapapillary region

In our study, assessment and measuring of the beta zone was based on the color photography of the fundus with centered on optic disc. Available optic papilla photographs of the right eyes were included in the study. After qualitative assessment, we recognized the beta zone and then measured it on the computer screen. The definition of beta zone is the area absence or marked atrophy of the retinal pigment epithelium (RPE), showed exposed sclera and visible choroidal macrovascular, located around to the optic papilla border [7]. After a training period, all of the selected photos were checked to distinguish the beta zone by one examiner (LXZ). If there had any queries, a panel (YXW and JBJ) would reassess the photographs. For those still with difficulties in recognition, the images of OCT were referenced. The microstructure of optic nerve and peripapillary connective tissues can be comprehensively displayed by the enhanced depth imaging (EDI) spectral-domain OCT (SD-OCT). Using the Image J software (version 1.49v; National Institutes of Health, USA; http://imagej.nih.gov/ij), the topographic parameters of the beta zone were measured respectively, including area, the circumferential angle, maximum radial extent (location), the distance between point of maximum radial extent and optic disc border (Fig. 1). Four sectors constitute the area of optic disc in clinical setting: the inferior temporal and superior temporal sectors were 90° and their middle lines were tilted 13° temporal to the vertical optic disc axis. Sector temporal was 64°covered temporal area, and the sector nasal was 116° covered the nasal area [3] (Fig. 2).

### Statistical analysis

We used SPSS (SPSS for Windows, version 22.0, IBM-SPSS Inc. Chicago, IL, USA), a commercially available statistical software package, for statistical analysis. Only the right eye was statistically analyzed. First of all, we calculated the frequency of the beta zone, determined position of the beta zone through which sector the maximum radial extent was located, then the data of area and the circumferential angle were given as mean ± standard deviation. In the next step, a univariate logistic regression analysis was performed, with presence of beta zone and area of beta zone as dependent parameter, ocular and general parameters as independent parameters. A multivariate logistic regression analysis was performed in the last step, with the presence of beta zone and area of beta zone as dependent parameter and all those parameters as independent parameters which were significantly associated with the beta zone in univariate analysis. We used the correlation coefficient *r* or *r*^*2*^ for reporting the statistical strength of correlations. All *P*-values were bilateral and statistical significance existed only when the *P*-values were lower than 0.05.

## Results

Our study is the third time follow-up of the Beijing Eye Study, out of the 4403 subjects included at baseline, 3468 (1963 (56.6%) women) said they would take part in the examination, therefore, the response rate is 78.8%. The average age of all subjects was 64.6 ± 9.8 years (median age: 64 years; range from 50 to 93 years). Out of the 3468 participants, the optic disc photographs of right eyes reaching the standard of reading were available for 3406 (98.2%) eyes (1921 (56.4%) women). The mean age was 64.5 ± 9.8 years (median: 63 years; range: 50 to 93 years), the mean refractive error (spherical equivalent) was - 0.20 ± 2.03 diopters (median: 0.25 diopters; range: − 22.0 to + 7.0 diopters). For 62 (1.8%) eyes, the optic disc photographs could not be measured either because photographs were not taken or because available photographs could not be assessed owing to keratoleukoma, lens opacities or vitreous clouding, et al.

The beta zone was found in 1358 (39.9% ± 1.6%) eyes. Mean age of all subjects with the beta zone was 67.3 ± 9.8 years (median: 68, range, 50–93 years), mean refractive error was − 0.66 ± 2.59 diopters (median: 0.0 diopter, range, − 20.0 to + 6.0 diopter). Area of the beta zone measured 0.37 ± 0.84 mm^2^, (median: 0.0, range, 0.0 to 14.98 mm^2^). The circumferential angle (width) of the beta zone was 203.5 ± 81.8° (median: 174.35°, range, 50.1 to 360.0°). The maximum radial extent was 0.36 ± 0.27 mm (median: 0.36 mm, range, 0.12 to 2.47 mm), was significantly longest in the temporal peripapillary region (883/1358 or 65.0%), followed by the temporal inferior region (297/1358 or 21.9%), the temporal superior region (119/1358 or 8.8%), and finally the nasal region (59/1358 or 4.3%).

In univariate analysis, the presence of beta zone significantly related or unrelated to the systemic parameters and ocular parameters were shown in the Table 1; the size of the beta zone significantly related or unrelated to the systemic parameters and ocular parameters were shown in the Table 2.

**Table 1:** Factors Associated with the presence of beta zone in the Beijing Eye Study (Univariate Analysis)

Parameter	***P***-Value	Odds Ratio	95% Confidence Interval	
Age (Years)	<0.001	1.051	1.043	1.059
Gender (men / Women)	<0.001	0.602	0.524	0.692
Rural / Urban Region of Habitation	<0.001	1.185	1.142	1.231
Body Mass Index (kg/m^2^)	<0.001	0.954	0.937	0.972
Level of Education (1–5)	<0.001	1.139	1.063	1.220
Self-Reported Income	<0.001	1.119	1.084	1.156
Cognitive Score	0.186	0.987	0.967	1.007
Alcohol Consumption Frequency	0.765	0.994	0.955	1.034
Smoking Never / Ever	0.549	1.048	0.899	1.222
Smoking Package Years	0.941	1.001	0.964	1.040
Hours Spent With Vigorously Intensive Physical Activities Per Week	0.824	0.991	0.913	1.075
Hours Spent With Moderate Physical Activities Per week	<0.001	0.915	0.890	0.941
Number of Hours Spent With Walking Per Week	0.109	1.026	0.994	1.058
Number of Hours Spent With Sitting Per Week	0.202	1.018	0.990	1.047
Hypertension history	0.436	1.060	0.916	1.225
Cardiovascular disease history	0.009	1.281	1.063	1.542
Diabetes history	0.705	0.959	0.770	1.194
Systolic Blood Pressure (mmHg)	0.798	1.000	0.996	1.003
Diastolic Blood Pressure (mmHg)	<0.001	0.988	0.983	0.994
Mean Blood Pressure (mmHg)	0.180	0.987	0.967	1.006
Glucose (mmol/L)	0.020	0.932	0.878	0.989
Cholesterol (mmol/L)	0.080	0.926	0.849	1.009
Creatinine	<0.001	1.011	1.005	1.017
High-Density Lipoproteins (mmol/L)	0.219	1.142	0.924	1.411
Low-Density Lipoproteins (mmol/L)	0.022	0.894	0.812	0.984
Triglycerides (mmol/L)	0.044	0.926	0.859	0.998
Use of Aspirin Yes / No	0.255	1.093	0.938	1.275
Depression Score	0.674	0.998	0.987	1.009
**Ophthalmological Parameters**				
Visual Acuity	<0.001	0.341	0.273	0.427
BCVA(LogMAR)	<0.001	2.291	1.543	3.400
Axial Length (mm)	<0.001	1.650	1.532	1.778
refractive errors	<0.001	0.825	0.793	0.858
Anterior Corneal Curvature Radius (mm)	<0.001	2.387	1.792	3.180
Central Corneal Thickness (μm)	0.007	1.003	1.001	1.005
Anterior Chamber Depth (mm)	<0.001	1.466	1.265	1.698
Lens Thickness (mm)	<0.001	1.579	1.264	1.973
Intraocular Pressure mmHg)	0.908	0.998	0.973	1.024
Retinal Nerve Fiber Layer Thickness (μm)	<0.001	0.964	0.958	0.970
Subfoveal Choroidal Thickness (μm)	<0.001	0.993	0.992	0.993
Fundus Tessellation	0.666	1.151	0.608	2.182
Macular Retinal Thickness (μm)	0.277	0.998	0.996	1.001
Optic Disc Size (mm^2^)	<0.001	1.756	1.491	2.068
Nuclear Cataract	<0.001	1.201	1.121	1.288
Cortical Cataract	0.240	1.369	0.811	2.310
Subcapsular Posterior Cataract	0.104	0.113	0.008	1.563
Glaucoma history	0.012	1.727	1.126	2.648
Glaucoma prevalence	<0.001	2.538	1.882	3.424
Age-Related Macular Degeneration, Prevalence, Total	0.006	0.819	0.710	0.944
Age-Related Macular Degeneration, Early Stage	0.428	0.902	0.699	1.164
Age-Related Macular Degeneration, Intermediate Stage	0.010	0.783	0.650	0.943
Age-Related Macular Degeneration, Late Stage	0.145	1.740	0.825	3.669
Diabetic Retinopathy, Prevalence	0.787	1.093	0.572	2.091
Retinal Vein Occlusion, Total	0.052	1.500	0.997	2.256
Polypoidal Choroidal Vasculopathy	0.120	2.158	0.819	5.683

**Table 2:** Factors Associated with area of the presence of beta zone in the Beijing Eye Study (Univariate Analysis)

Parameter	***P***-Value	Standardized Coefficient Beta	Regression Coefficient B	95% Confidence Interval
Age (Years)	<0.001	0.236	0.020	0.017, 0.023
Gender (men / Women)	0.006	−0.047	− 0.079	− 0.136, − 0.022
Rural / Urban Region of Habitation	<0.001	0.149	0.067	0.052, 0.082
Body Mass Index (kg/m2)	<0.001	−0.097	− 0.021	− 0.028, − 0.013
Level of Education (1–5)	0.037	0.037	0.029	0.002, 0.056
Self-Reported Income	<0.001	0.113	0.042	0.029, 0.055
Cognitive Score	<0.001	−0.064	− 0.014	− 0.021, − 0.006
Alcohol Consumption Frequency	0.010	− 0.045	− 0.021	− 0.037, − 0.005
Smoking Never / Ever	0.049	− 0.035	− 0.063	− 0.125, 0
Smoking Package Years	0.069	−0.032	− 0.014	− 0.029, 0.001
Hours Spent With Vigorously Intensive Physical Activities Per Week	0.417	0.015	0.014	− 0.019, 0.046
Hours Spent With Moderate Physical Activities Per week	<0.001	− 0.108	−0.030	− 0.040, − 0.021
Hours Spent With Walking Per Week	0.020	0.042	0.015	0.002, 0.027
Hours Spent With Sitting Per Week	0.873	0.003	0.001	−0.010, 0.012
Hypertension history	0.013	0.045	0.076	0.016, 0.135
Cardiovascular disease history	<0.001	0.088	0.188	0.112, 0.264
Diabetes history	0.434	0.015	0.035	−0.053, 0.123
Systolic Blood Pressure (mmHg)	0.347	0.016	0.001	−0.001, 0.002
Diastolic Blood Pressure (mmHg)	<0.001	−0.077	−0.005	− 0.007, − 0.003
Mean Blood Pressure (mmHg)	0.699	− 0.023	− 0.001	− 0.005, 0.003
Glucose (mmol/L)	0.050	− 0.040	−0.019	− 0.037, 0
Cholesterol (mmol/L)	0.380	−0.018	− 0.013	− 0.042, 0.016
Creatinine	0.005	0.071	0.002	0.001, 0.003
High-Density Lipoproteins (mmol/L)	0.341	0.019	0.035	−0.037, 0.107
Low-Density Lipoproteins (mmol/L)	0.229	−0.025	−0.020	− 0.052, 0.013
Triglycerides (mmol/L)	0.104	−0.033	−0.020	− 0.044, 0.004
Use of Aspirin Yes / No	0.011	0.045	0.080	0.018, 0.142
Depression Score	0.256	0.020	0.003	−0.002, 0.007
**Ophthalmological Parameters**				
Visual Acuity	<0.001	−0.247	− 0.647	− 0.732, − 0.562
BCVA(LogMAR)	<0.001	0.168	0.748	0.600, 0.896
Axial Length (mm)	<0.001	0.385	0.261	0.239, 0.283
refractive errors	<0.001	−0.387	−0.155	−0.168, − 0.142
Anterior Corneal Curvature Radius (mm)	<0.001	0.087	0.263	0.157, 0.369
Central Corneal Thickness (μm)	0.012	0.045	0.001	0, 0.002
Anterior Chamber Depth (mm)	<0.001	0.081	0.124	0.070, 0.178
Lens Thickness (mm)	<0.001	0.090	0.198	0.120, 0.277
Intraocular Pressure mmHg)	0.371	−0.016	−0.005	− 0.015, 0.006
Retinal Nerve Fiber Layer Thickness (μm)	<0.001	−0.256	−0.017	− 0.019, − 0.014
Subfoveal Choroidal Thickness (μm)	<0.001	− 0.367	− 0.003	− 0.003, − 0.002
Fundus Tessellation	0.149	− 0.025	−0.190	− 0.447, 0.068
Macular Retinal Thickness (μm)	0.878	0.003	0.0000747	−0.001, 0.001
Optic Disc Size (mm2)	<0.001	0.097	0.152	0.091, 0.213
Nuclear Cataract	<0.001	0.117	0.075	0.051, 0.099
Cortical Cataract	<0.001	0.106	0.515	0.333, 0.698
Subcapsular Posterior Cataract	0.851	0.004	0.065	−0.616, 0.746
Glaucoma history	<0.001	0.078	0.372	0.198, 0.547
Glaucoma prevalence	<0.001	0.148	0.535	0.415, 0.655
Age-Related Macular Degeneration, Prevalence, Total	0.004	−0.050	−0.085	−0.142, 0.028
Age-Related Macular Degeneration, Early Stage	0.950	−0.001	−0.003	− 0.107, − 0.100
Age-Related Macular Degeneration, Intermediate Stage	0.002	− 0.054	−0.118	− 0.193, 0.044
Age-Related Macular Degeneration, Late Stage	0.218	0.021	0.195	−0.116, 0.506
Diabetic Retinopathy, Prevalence	0.017	0.044	0.304	0.053, 0.555
Retinal Vein Occlusion, Total	0.215	0.021	0.104	−0.060, 0.268
Polypoidal Choroidal Vasculopathy	0.022	0.039	0.455	0.065, 0.846

In the multivariate analysis, all factors with statistical significance in the univariate associations (Tables 1 and 2) were considered as the starting points of our model construction. From this full model, we used a step-wise manner to remove non-significant terms, beginning with the parameters that have the highest *P*-values. Finally, the presence of beta zone has statistically significant association with male gender (*P* = 0.001), myopic ametropia (*P* = 0.003), thinner retinal nerve fiber layer thickness (*P*<0.001), thinner subfoveal choroidal thickness (*P*<0.001), bigger size of optic disc size (*P*<0.001) (Table 3). The size of beta zone has statistically significant association with increasing age (*P*<0.001), urban (*P* = 0.025), cardiovascular disease history (*P* = 0.025), longer axial length (*P* = 0.004), myopic refractive error (*P*<0.001), thinner retinal nerve fiber layer thickness (*P* = 0.001), thinner subfoveal choroidal thickness (*P*<0.001), bigger size of optic disc size (*P* = 0.001), age related macular degeneration (*P* = 0.038) (Table 4).

**Table 3:** Factors Associated with the presence of beta zone in the Beijing Eye Study (Multivariate Analysis)

Parameter	***P***-Value	Odds Ratio	95% Confidence Interval	
Gender (men / Women)	0.001	0.548	0.384	0.782
Refractive errors	0.003	0.856	0.774	0.948
Retinal Nerve Fiber Layer Thickness (μm)	<0.001	0.969	0.952	0.986
Subfoveal Choroidal Thickness (μm)	<0.001	0.992	0.990	0.994
Optic Disc Size (mm^2^)	<0.001	2.628	1.787	3.865

**Table 4:** Factors Associated with area of the presence of beta zone in the Beijing Eye Study (Multivariate Analysis)

Parameter	***P***-Value	Standardized Coefficient Beta	Regression Coefficient B	95% Confidence Interval
Age (Years)	<0.001	0.242	0.018	0.012, 0.024
Rural / Urban Region of Habitation	0.025	−0.089	−0.043	− 0.080, − 0.005
Cardiovascular disease history	0.025	− 0.085	− 0.141	− 0.265, − 0.018
Axial Length (mm)	0.004	0.141	0.084	0.026, 0.142
refractive errors	<0.001	−0.245	−0.087	− 0.121, − 0.053
Retinal Nerve Fiber Layer Thickness (μm)	0.001	− 0.125	−0.007	− 0.012, − 0.003
Subfoveal Choroidal Thickness (μm)	<0.001	− 0.154	−0.001	− 0.002, 0
Optic Disc Size (mm^2^)	0.001	0.125	0.168	0.072, 0.263
Age-Related Macular Degeneration, Prevalence, Total	0.038	−0.074	− 0.099	−0.192, 0.006

## Discussion

Peripapillary atrophy has always been considered as a possible risk factor related to glaucoma progression since found in the twentieth century [13]. Histological and radiographic evidences demonstrate that significant disappearance of photoreceptor cells and retinal pigment epithelial cells as well as occlusion of choroid capillary is the main causes of formation of the beta zone [14]. Studies have confirmed that the peripapillary atrophy zone has correspondence in space with the most obvious visual field damage. At present, direct ophthalmoscope, indirect ophthalmoscope, slit lamp with pre-set lens and fundus stereoscopic photography are commonly used to observe the beta zone. These detection methods are convenient and fast, but with poor reliability and accuracy. In recent years, along with the enhancement of computer image processing technology, researchers can take advantage of the computer image processing softwares, such as photoshop and Image J to outline the boundary of optic disc and peripapillary atrophy zone as well as the angle and line that need to be measured. Then, quantitative measurement of these parameters can be more accurate to study the morphology of peripapillary atrophy zone and the results are more credible [15, 16]. The population prevalence of the beta zone was 15 to 20% according to a report [5]. It was easy to appear in the temporal region with the largest atrophy area, followed by the temporal inferior region and temporal superior region, and at least the nasal region with the minimal atrophy area.

In this study, we found that the population prevalence of beta zone was 39.9, which was a significant increase compared to the past prevalence study and the Beijing Eye Study in 2001 (20.95%) [7]. This may be due to the following reasons. First, the age of Beijing Eye Study population was older as a whole (over 50 in 2011), and this number increased significantly because the occurrence and development of the beta zone have obvious relationship with age. Second, the previous studies have determined beta zone only by the subjective judgment of an ophthalmologist, so it was hard to ascertain the beta zone when it was small, atypical, or the refractive media was not clear. In this study, SD-OCT with enhanced depth imaging was used to evaluate the fundus for the first time. Clinical and scientific research workers can observe the fault structures of peripapillary retina and choroid better, so they can make a clearer judgment and positioning of beta zone to make the results more reliable and credible. In this study, the beta zone of all the images was further evaluated by comparing with SD-OCT images when the digital photos cannot be determined by human or questioned. It can be speculated that former researchers were likely to ignore or mistaken part of the beta zone for alpha zone, resulting in the previous lower population prevalence of the beta zone.

In this study, the distribution of the beta zone both in the present and previous research was consistent, namely easy to appear in the temporal region with the largest atrophy area, followed by the temporal inferior region and temporal superior region, and at least the nasal region with the minimal atrophy area. With the development of OCT technology, some scholars further use EDI-OCT to call the area which is closer to the optic papilla and lack of Bruch’s membrane as gamma zone, and this area was considered to be related with absence of glaucoma, age, and refractive status [17]. The combination of gamma zone can enhance the performance of the diagnosis and prediction of glaucoma with the beta zone. However, gamma zone is very difficult to distinguish without OCT.

In this study, the size of the beta zone has statistically significant association with increasing age, urban, cardiovascular disease history, axial length, myopic refractive error, retinal nerve fiber layer thickness, subfoveal choroidal thickness, optic disc size, age related macular degeneration. In Beijing Eye Study in 2001, the average age of the study population was 55. We found that age and the size of the beta zone were significantly related, and the area enlarged about 0.21 mm [2] every 10 years [7]. In 2006, the population follow-up found that the progress rate of the size of the beta zone was 8.2 ± 0.5% [8]. In 2011, the population was at an average age of 64 years, and the population prevalence of the beta zone was quite different from the previous study, so the data was not compared with that of 2001 and 2006. However, the population prevalence of the beta zone in this study was much higher than that of other population-based studies [4–6], and age was probably one of the important influencing factors. Changes in histological structure may lead to decreased blood supply of the atrophy zone or blood-optic disc barrier dysfunction [18]. Cardiovascular disease is a large group of diseases, some of them may cause eye ischemia and hypoxia which can lead to degenerate of RPE. So the larger atrophy area with less blood supply may cause enlargement of the atrophy zone easily [19]. But in some previous study, central BP has no significant effect on parapapillary atrophy. More accurate predictors of tissue perfusion are still needed to determine blood flow at the level of the optic nerve head [20]. In our study, we did not conduct separate statistical analyses for this group of diseases, so we cannot draw any major conclusion from this. Further studies are needed to determine which cardiovascular diseases are associated with beta zone. Prior studies have in-depth research on beta zone in high myopia [21, 22]. They found that peripapillary scleral ring broadened and the beta zone enlarged obviously in patients with high myopia compared to those without high myopia. The optic disc area increased by 1.6%, the disc edge area increased by 1.4% and the beta zone area increased by 1.3% when myopia increased a diopter [4]. In our study, the beta zone area is related to axial length, myopic ametropia and optic pallia size, which is consistent with previous studies. Imamura believed that the continuous growth of axial length causes the mechanical stretching and thinning of choroid and RPE, which is one of the main causes of the atrophy of choroid and retinal pigment epithelium [23]. Therefore, the association of the existence of beta zone with myopia should be considered when the beta zone is used as the diagnosis indicator of glaucoma. However, the beta zone in presbyopia is relatively rare, so the beta zone found in presbyopia may predict an increased possibility of glaucoma. The histological character of the beta zone and age-related macular degeneration (AMD) is similar, both presenting retinal pigment epithelium layer degeneration and choroid capillary occlusion to cause the loss of retinal pigment epithelial cells. The results of Beijing Eye Study in 2001 demonstrated that the beta zone correlated with AMD in univariate analysis, but their correlation was not obvious in multivariate analysis. However, this study found that the smaller size of beta zone was significantly related with macular degeneration, may be because the age is a related factor of beta zone and AMD, and whether the existence of the beta zone would increase the risk of AMD is a meaningful research direction in the future. By the adjuvant of SD-OCT, SFCT was measured in this study for the first time, and we found that the beta zone and the SFCT could be relevant. The variation of the choroid thickness is very important for the accurate assessment of many diseases. The thickness of the choroid depends on choroidal blood perfusion, and the important factor of the occurrence of the beta zone is peripapillary poor choroidal perfusion. However, the change sequence of the beta zone and SFCT is not certain now, and research on their relationship has not been carried out yet. The causal relationship between them also needs long time follow-up and shorter follow-up interval to prove.

At present, the research on beta zone is mainly in the field of glaucoma. Certainly, the prevalence and the size of the beta zone of the patients with glaucomatous optic nerve injury are significantly greater than that of the normal eyes, and larger size of the beta-zone at baseline is the risk factor of the beta-zone expansion [13]. Changes in histological structure may lead to decreased blood supply of the atrophy zone or blood-optic disc barrier dysfunction [24]. Larger atrophy area with less blood supply may cause enlargement of the atrophy zone easily [25]. In the initial diagnosis of glaucoma, the disease progression was faster if there are beta zone in patients’ fundus, therefore, with the beta zone and its development is the important parameter of glaucoma. In our study, the presence and the area of the beta zone has statistically significant association with the glaucoma in univariate analysis, but the significant association does not exist in multivariate analysis. The reason might be that the subjects were different, and most of previous studies were hospital-based studies. In the normal people, the glaucoma might not be the main cause of the beta zone changes. Another possible reason is that the inclusion of retinal nerve fiber layer thickness in the analyses. It is well known that retinal nerve fiber layer thickness correlates highly with glaucoma.

There are still some insufficiencies of this study. The Beijing Eye Study 2011 is the third time follow up in the population, non-participation is the major concern. The response rate was 78.8%, which is reasonable, however, a selection bias may be caused by the differences between participants and nonparticipants. The assessment of the beta zone was done primarily by one examiner, there may be potential biases. Our study was based on fundus photographs, the size of beta zone was the result of actual measurement without consideration of magnification effect. Participants cannot be taken available fundus photographs for opacity of refracting media, which is also a limiting factor of this study. The measurement figures for peripapillary atrophy may be artificially underestimated because of it. Moreover, findings from our study cannot allow to directly drawing conclusions on cause-relationships. Strengths of our study are that SD-OCT with enhanced depth imaging was used for the first time in a population-based prevalence study, and made the results more reliable and credible.

## Conclusion

In conclusion, the population prevalence of beta zone was 39.9% in elderly Chinese. The beta zone most often appeared in the temporal peripapillary region, followed by the temporal inferior region, the temporal superior region, and finally the nasal region, and the size of beta zone was associated with age, urban, cardiovascular disease history, axial length, myopic refractive error, retinal nerve fiber layer thickness, subfoveal choroidal thickness, optic disc size, age related macular degeneration**.**

## Data Availability

The datasets generated and analysed during the current study are not publicly available due to some of the data are pending patent application, but are available from the corresponding author on reasonable request.
